# No ancillary proteins are essential for the functional heterologous expression of the homomeric α6 nicotinic acetylcholine receptor from the brown castor tick *Ixodes ricinus*

**DOI:** 10.1016/j.crpvbd.2026.100430

**Published:** 2026-09-04

**Authors:** Muhammad Tanveer Khan, Kiranpreet Kaur, Tor Einar Horsberg, Marit Jorgensen Bakke

**Affiliations:** a Norwegian University of Life Sciences, Faculty of Veterinary Medicine, Aas, Norway; b Aker Qrill Company, Lysaker, Norway

**Keywords:** *Ixodes ricinus*, Nicotinic acetylcholine receptor, Functional expression, Ancillary proteins, Acetylcholine

## Abstract

Nicotinic acetylcholine receptors (nAChRs) are ligand-gated ion channels that facilitate rapid neural signaling. nAChRs respond to acetylcholine, their natural agonist, as well as to nicotine and neonicotinoids, the latter used to control ectoparasites and agricultural pests. The nAChRs are well studied in mammals and some arthropods; however, the nAChR subunits in the ectoparasitic tick *Ixodes ricinus* remain less understood. This study examined the α6 nAChR subunit in *I*. *ricinus* at both the molecular and functional levels. RT-PCR identified two alternative splice variants of the α6 subunit: *Iricα6A*, which includes two variants of exon 3 (3a and 3b) and *Iricα6B*, which contains only 3b. Protein structure predictions identified a loop of 15 amino acids within the extracellular domain of Iricα6A, originating from exon 3a, that is missing in Iricα6B; however, the role of this loop in receptor non-functionality remains undetermined. In functional studies using *Xenopus laevis* oocytes, two-electrode voltage-clamping revealed that only Iricα6B forms a functional homomeric receptor and does not require ancillary proteins. The Iricα6B EC_50_ for ACh was estimated to be 2 μM (*n* = 7 different recordings from two different batches of oocytes). The neonicotinoids dinotefuran and imidacloprid exhibited varying potencies and efficacies, with EC_50_ values of 0.42 nM (*n* = 6 different recordings from two different batches of oocytes) and 11.6 nM (*n* = 6 different recordings from two different batches of oocytes), respectively, and maximum amplitudes of 112% and 81% relative to ACh, respectively. Nicotine and two additional neonicotinoids also elicited responses in the Iricα6B receptor.

## Highlights

• *Ixodes ricinus* α6 subunit of nicotinic acetylcholine receptor has two mRNA variants.
• Both mRNA variants are present in different developmental stages of *I. ricinus.*
• Only one variant forms functional homomeric receptors in *Xenopus laevis* oocytes.
• First expression of a functional arthropod α6 receptor without ancillary proteins.
• The α6 receptor responds to acetylcholine and different neonicotinoids.

## Graphical abstract

**Figure:**
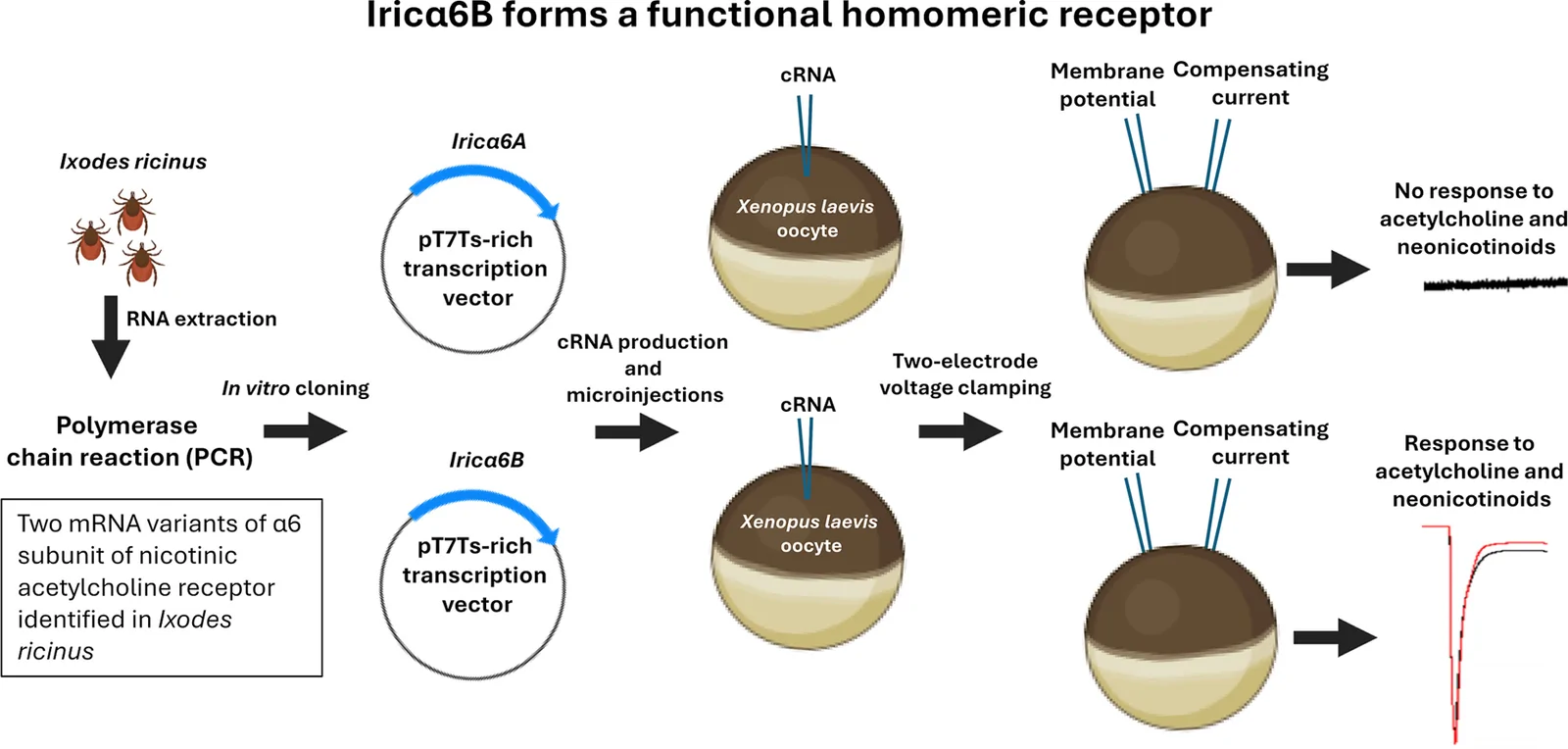

## 1 Introduction

Ticks, belonging to the class Arachnida, are obligatory parasites that feed on their host’s blood for growth and survival. They are considered a major public health and veterinary issue worldwide. Ticks directly impact livestock production by depleting blood, irritating and suppressing immune functions, reducing milk and meat production and destroying leather quality (Desta, 2016; Tabor et al., 2017; Obaid et al., 2022). Indirect losses result from the transmission of tick-borne pathogens to humans and animals (Desta, 2016), including tick-borne encephalitis and Lyme borreliosis in humans, and babesiosis and theileriosis in animals (Jongejan and Uilenberg, 2004; Anderson and Magnarelli, 2008; Lindquist, 2008; Barbour and Benach, 2019). A variety of substance classes, including organophosphates, pyrethroids and macrocyclic lactones, are used against ticks (George et al., 2004). However, numerous limitations are associated with their use, including resistance development, environmental contamination, and drug residues in food (Abbas et al., 2014; Tabor et al., 2017; Obaid et al., 2022). Moreover, vaccines against ticks are a promising alternative to acaricides, but are not sufficient to provide full protection against multiple species of ticks in different parts of the world (de la Fuente and Contreras, 2015; de la Fuente et al., 2016; Schetters et al., 2016). The animal health industry invests substantial amounts annually in the research and development of new antiparasitic drugs, with tick-targeting products accounting for a significant share of this investment (Woods and Williams, 2007).

Nicotinic acetylcholine receptors (nAChRs) are primarily located in the postsynaptic membrane of cholinergic synapses and function as ligand-gated ion channels. nAChRs are activated by the neurotransmitter acetylcholine and by substances such as nicotine (Karlin, 2002) and neonicotinoids, which are widely used to combat ectoparasites and agricultural pests. Despite their importance, detailed studies of the function of these channels in arthropods are limited due to challenges in reconstituting functional nAChRs containing both α and β subunits solely from genes of the investigated arthropod in an *ex vivo* system (Karlin, 2002; Matsuda et al., 2020).

Functional nAChRs are homo- or heteropentameric complexes composed of homologous subunits, forming an ion channel (Jones and Sattelle, 2010). Subunits are classified into alpha (α) and non-alpha (β, γ, δ, and ε) based on whether they possess two adjacent cysteine residues in loop C or not, respectively (Kao and Karlin, 1986). Each subunit has an extracellular N-terminal domain featuring a cys-loop with two cysteine residues separated by 13 amino acids, four transmembrane segments (TM1-TM4), and an intracellular loop essential for receptor function and localization (Kao and Karlin, 1986). The acetylcholine (ACh) binding site, which is vital for receptor activation, is situated between neighbouring subunits and comprises six distinct regions (Holden-Dye et al., 2013). Homopentameric nAChRs consist exclusively of alpha subunits, whereas heteropentamers are composed of various combinations of alpha and non-alpha subunits (Grauso et al., 2002; Rufener et al., 2020; Hawkins et al., 2022). In vertebrates, 17 nAChR subunits have been identified (Millar, 2003). For instance, mammalian α7, α9 and α10 can form homopentameric nAChRs (Elgoyhen et al., 1994; Tekarli et al., 2025). In contrast, combinations of α2-α7 and β2-β4 nAChR subunits can assemble into diverse heteropentameric receptor subtypes with distinct subunit compositions and stoichiometries, further increasing the complexity of nAChR diversity. For example, α4 and β2 form two receptors, (α4)_2_(β2)_3_ and (α4)_3_(β2)_2_, with high and low affinity for ACh, respectively (Nelson et al., 2003).

In comparison, arthropods have fewer nAChR genes. However, receptor diversity can still be significantly increased through mechanisms such as alternative splicing, cassette exons and A-to-I RNA editing, which provide the molecular basis for a broad functional diversity (Seeburg, 2002; Sattelle et al., 2005; Rinkevich and Scott, 2009; Jones and Sattelle, 2010). In arthropods, both homopentameric and heteropentameric nAChRs have been identified, as in mammals. The functional demonstration of a homomeric insect nAChR in *Xenopus laevis* oocytes was reported in *Drosophila melanogaster*, where the α5 and α7 subunits formed functional homomeric receptors when co-expressed with RIC-3 (Lansdell et al., 2012). *Xenopus laevis* oocytes are widely used as a heterologous expression system to study pharmacological properties of ion channels (Kusano et al., 1977). However, the heterologous expression and functional characterization of heteropentameric nAChRs from invertebrates in *X. laevis* oocytes remain challenging, particularly for receptors composed exclusively of invertebrate-derived subunits. Some progress has been made with arthropod channels by creating hybrid receptors that combine the α-subunit from the target species with a β-subunit from a chicken (Bertrand et al., 1994). The first non-hybrid arthropod nAChRs to be functionally expressed in *X. laevis* oocytes were identified in the salmon louse, *Lepeophtheirus salmonis*. These included Lsa-nAChR1, comprising α1, α2, β1, and β2 subunits, and Lsa-nAChR2, comprising α3, β1, and β2 subunits, which exhibited distinct properties (Rufener et al., 2020).

It has been difficult to express the nAChR subunits as functional receptors in the *X*. *laevis* oocyte system. In ticks, the first nAChR subtype was isolated from the brown dog tick, *Rhipicephalus sanguineus,* and subsequently functionally characterized in *X. laevis* oocytes as a chimeric receptor (Lees et al., 2014). The nAChR α subunit alone (*Rsanα1*) failed to express a homomeric receptor in the presence or absence of *Caenorhabditis elegans* RIC3 or *X. laevis* RIC3. However, when co-expressed with the chicken β2 nAChR subunit, ACh evoked concentration-dependent inward currents, and the resulting hybrid receptor was also sensitive to nicotine and choline. Interestingly, the hybrid receptor was insensitive to imidacloprid and spinosad. Tick nAChRs have also been characterized using microtransplantation of synganglion membrane preparations into *X. laevis* oocytes, revealing responses to ACh, nicotine and neonicotinoids (Le Mauff et al., 2020). In parallel with the present study, Cartereau and colleagues also managed to express the α6 subunit from ticks as a homomeric receptor in *X*. *laevis* oocytes, both with chaperone proteins from *C*. *elegans* and *Rattus norvegicus* and without chaperone proteins (Cartereau et al., 2025). Transcriptomic analyses of the *I*. *ricinus* synganglion identified eight genes encoding nAChR subunits, including the α6 subunit (Rispe et al., 2022). The α6 nAChR subunit is related to the α7 subunit (Thany et al., 2007), which is known to form functional homomeric receptors in *D*. *melanogaster* when heterologously expressed in *X. laevis* oocytes (Lansdell et al., 2012). Two major insecticide classes, the neonicotinoids and spinosyns, target the nAChRs, with spinosyns believed to target the insect α6 nAChR subunit specifically, unlike neonicotinoids (Ffrench-Constant et al., 2016; Perry et al., 2021; Lu et al., 2022). However, whether the α6 nAChR subunit of ticks plays a comparable role in sensitivity to these insecticide classes remains largely unexplored.

Here, we selected the α6 subunit from *I. ricinus* to determine its ability to form a functional homomeric receptor when expressed in *X. laevis* oocytes, either with or without *I. ricinus*-specific ancillary proteins, and to characterize its pharmacological sensitivity to neonicotinoids, thereby providing insights that may contribute to the development of more selective acaricides.

## 2 Materials and methods

### 2.1 Ticks and RNA extraction

The three developmental stages (larvae, nymphs and unfed adults (males and females)) of castor bean ticks (*Ixodes ricinus*) were purchased from IS Insect Services, Germany. As soon as tick samples were received, they were placed on dry ice for rapid freezing and stored at −80 °C until further processing. RNA was extracted from pooled groups of larvae (*n* = 50), nymphs (*n* = 25), adult females (*n* = 2) and males (*n* = 2). Thus, one independent RNA extraction was performed from each developmental stage of *I*. *ricinus,* and the resulting RNA was used for RT-PCR and cloning. Tick samples were placed in 1.5 ml Eppendorf tubes with 1 ml of TRIzol reagent (Sigma-Aldrich, St. Louis, MO, USA), a monophasic solution of phenol and guanidine isothiocyanate that facilitates tissue lysis and protects the RNA from degradation. The samples were then homogenized in two steps. First, the tick samples were manually crushed with a pestle. During crushing, the samples were placed on dry ice several times to prevent overheating. The process took approximately 5 min before the tick samples were semi-homogenized. In the second step, the rough homogenates were placed in 2 ml Precellys (Bertin Technologies, Montigny-le-Bretonneux, France) tubes with a 5-mm stainless steel bead and homogenized by running 2–3 cycles of 15 s at 5000–6000 *rpm*. The tubes were placed on dry ice between cycles to prevent overheating. The thoroughly homogenized samples were incubated in 0.2 ml chloroform (Sigma-Aldrich) for 3 min, then centrifuged for 15 min at 12,000× *g* at 4 °C to facilitate phase separation. After carefully transferring the aqueous phase to spin columns supplied with the RNeasy Micro kit (Qiagen, Hilden, Germany), RNA was isolated and purified as instructed. The concentration of RNA in the eluted samples was determined by measuring the absorbance at 260 nm (A260) using a spectrophotometer (Epoch, BioTek Instruments, Winooski, VT, USA). The purity was assessed by calculating the ratios of absorbance at 260 nm to 280 nm (A260/A280) and at 260 nm to 230 nm (A260/A230), with typical values of 1.8–2.1. The integrity of the RNA was verified by 1% agarose gel electrophoresis in 1× TBE (Tris-borate-EDTA) buffer, with SYBR Gold nucleic acid stain (Thermo Fisher Scientific, Waltham, MA, USA) used to visualize the RNA.

### 2.2 Identification and cloning of *Iricα6* subunit and ancillary proteins

The full-length *I. ricinus* nAChR alpha 6 (*Iricα6*) mRNA sequence was obtained from the NCBI GenBank database (MZ027288.1). For other nAChR receptors from arthropods, co-expression of ancillary proteins has been necessary (Rufener et al., 2020; Komori et al., 2023). Thus, these were also obtained for *I. ricinus* from NCBI GenBank (*UNC-50*: GFVZ01133238.1; *UNC-74*: GFVZ01112425.1; *RIC3*: GFVZ01112425.1).

Primers for cloning genes into pT7TS-rich transcription vectors (Addgene plasmid #99050) were designed using Primer-BLAST with restriction enzyme sites introduced in both forward and reverse primers (Supplementary Table S1). A one-step system (SuperScript IV One-Step RT-PCR System, Invitrogen, Carlsbad, CA, USA) that combines cDNA synthesis and PCR in a single reaction was used to produce gene-specific products. PCR amplification was performed accordingly: cDNA synthesis step at 55–60 °C for 10 min, pre-heating at 98 °C for 3 min, followed by 35 cycles of denaturation at 98 °C for 10 s, annealing at 60–68 °C for 10 s, extension at 72 °C for 1 min and the final extension step at 72 °C for 5 min. The PCR products were purified using QIAquick Gel Extraction Kit (Qiagen). The purified products were cloned into pT7TS-rich and transformed into MAX Efficiency™ DH5α competent cells (Invitrogen). Cells were incubated overnight at 37 °C on LB agar plates supplemented with 100 μg/ml of ampicillin. After cloning, the three to five positive plasmids were purified using the ZymoPURE Plasmid Miniprep Kit and the inserts were sequenced using LightRun (Eurofins Genomics, Ebersberg, Germany). The obtained forward and reverse sequences were assembled using Benchling (https://www.benchling.com/), and a sequence-verified plasmid was selected for each gene to synthesize the capped RNA. For multiple sequence alignment, BioEdit v.7.2.5 software was used (Hall, 1999).

### 2.3 Capped RNA (cRNA) synthesis

Templates for cRNA production consisted of PCR products amplified from pT7Ts-rich plasmids (primer pairs, Supplementary Table S1) containing complete cDNAs for the specific genes (Iricα6, *RIC3*, *UNC-50* and *UNC-74*), along with essential elements such as the T7 promoter, *X. laevis ß-*globin, and untranslated cDNA 5′- and 3′-ends of inserts necessary for *in vitro* transcription and protein expression in *X. laevis* oocytes. PCR products were amplified using Phusion High-Fidelity DNA polymerase (Thermo Fisher Scientific) with primer pairs listed in Supplementary Table S1. The following cycling conditions were used: initial denaturation at 98 °C for 30 s, followed by 30 cycles of denaturation at 98 °C for 10 s, annealing at 58 °C for 15 s, extension at 72 °C for 1 min, and a final extension step at 72 °C for 7 min. The PCR products were subjected to cRNA synthesis using the mMESSAGE mMACHINE™ T7 Transcription Kit (Thermo Fisher Scientific) according to the manufacturer’s instructions. The cRNA was resuspended in RNase-free water, and its concentration, quality, and integrity were assessed using spectrophotometry (Epoch, BioTek) and 1% RNA agarose gel electrophoresis as described above. Finally, the cRNA was aliquoted and stored at −80 °C until use.

### 2.4 Oocyte microinjection and electrophysiology

Defolliculated *X. laevis* oocytes were purchased from Ecocyte (Dortmund, Germany). Upon arrival, the oocytes were transferred to 96-well culture plates containing 200 μl of sterile Modified Barth’s solution (MBS) per well, with one oocyte per well. MBS contained NaCl (88 mM), KCl (1 mM), CaCl_2_ (0.4 mM), Ca (NO_3_)_2_ (0.33 mM), MgSO_4_ (0.8 mM), Tris-HCl (5 mM) and NaHCO_3_ (2.4 mM) at pH 7.4, and supplemented with 30 μg/ml, kanamycin sulfate, benzylpenicillin (100 Units/ml), streptomycin (100 μg/ml) and sodium pyruvate (2.5 mM). Oocytes were incubated at 18 °C for 2–3 h before injections on the same day or incubated overnight when injections were performed the following day. For microinjection, a Roboinject automatic injection system (Multi Channel Systems, Reutlingen, Germany) was used. Depending on the cRNA mixture, 3–15 ng of *Iricα6* was injected either alone or together with 3–15 ng of each ancillary cRNA (*RIC3*, *UNC-50* and *UNC-74*), in a total injection volume of 15–50 nl. After injections, the oocytes were incubated at 18 °C.

Functional expression and pharmacological responses were recorded 2–4 days post-cRNA injection using two-electrode voltage-clamp electrophysiology with the Roboocyte2 system (Multi Channel Systems, Reutlingen, Germany). During recordings, oocytes were clamped with two glass electrodes filled with 3 M KCl, and the membrane potential was maintained at −80 mV for acetylcholine experiments. However, when nicotine and neonicotinoids were tested, the responses were more stable at −100 mV than at −80 mV. Therefore, nicotine and neonicotinoids were tested at −100 mV, including the acetylcholine exposure performed immediately before testing these agonists. Oocytes were washed with oocyte normal frog Ringer (NFR) consisting of 90 mM NaCl, 2 mM KCl, 5 mM HEPES, 2 mM CaCl_2_, and 1 mM MgCl_2_ at pH 7.4, and experiments were carried out at 18 °C. All agonists were freshly prepared in NFR before the experiment. All recordings were performed using oocytes from at least two independent batches obtained from two different frogs for each agonist at varying concentrations. The curves and data obtained from Roboocyte 2 were evaluated in Roboocyte^2+^ (Multi Channel Systems). Dose-response curves were analyzed using the non-linear regression four-parameter (4 PL) logistic model with Prism software (GraphPad Software, Boston, MA, USA) according to the equation mentioned below. Responses to different agonists were normalized to the acetylcholine (300 μM) response recorded from the same oocyte before proceeding with the dose-response curves. Only individual oocytes that met the predefined quality criteria, including a stable resting membrane potential and reproducible responses to ACh, were included in the analysis, with 4–7 individual oocytes analyzed for each agonist. The normalized response Y was calculated as follows:

Y = I_min_ + (I_max_ – I_min_)/(1 + 10(log(EC_50_ – X)H))

where Y is the normalized response, I_max_ is the maximum response, I_min_ is the minimum response, H is the Hill coefficient, EC_50_ is the concentration that elicits half the maximum response, and X represents the logarithm of the compound concentration.

Responses of oocytes to two concentrations (1 μM and 100 μM) of nicotine, thiacloprid, and nitenpyram were assessed statistically using the Wilcoxon signed-rank test. Oocytes from two batches were used, and only those meeting the predefined quality criteria described above were included in the analysis. Responses to nicotine, thiacloprid, and nitenpyram were normalized to the ACh response recorded from the same oocyte immediately before agonist application. Oocytes from the two independent batches were analyzed together, with individual oocytes treated as experimental replicates.

### 2.5 Structural modelling and analysis

Protein 3D model predictions were conducted using a standard standalone version of AlphaFold 2, v.2.3.2 (Jumper et al., 2021) and its extension to multi-chain proteins, AlphaFold-Multimer (Evans, 2022). The modelling was run on Linux Ubuntu 22.04.5 on a Lenovo ThinkStation P720 equipped with an NVIDIA RTX A4000 graphics card, using the databases and default parameters as recommended in the GitHub repository of codes and databases (https://github.com/google-deepmind/alphafold). The predicted structures were analyzed using ChimeraX (Pettersen et al., 2021). However, it should be noted that AlphaFold models represent predicted protein structures and may not fully determine the flexibility of loop regions or accurately reflect the physiological conformation of the receptor. *In silico* receptor-ACh interaction studies were performed using AutoDock 4 (Huey et al., 2007) hosted on the online platform DockingServer (https://www.dockingserver.com/web). Docking results are interpreted as predictions that may help identify the potential ligand interacting with residues, rather than as definitive evidence of ligand binding. Two adjacent subunits were selected from the predicted pentameric structures of both variants and uploaded to DockingServer in PDB format. The ligand (ACh) was obtained from the PubChem database. After preparing the protein and ligand structures, molecular docking was performed using 100 independent runs, each terminated after a maximum of 2,500,000 energy evaluations. The population size was set to the default value of 150. During the docking search, the torsion step and the root-mean-square deviation (RMSD) tolerance steps were set to 0.2 Å and 2.0 Å, respectively. The rigid-body orientation and dihedral angle steps were set to 5°. The resulting docking poses were downloaded in PDB format and subsequently analyzed using ChimeraX.

## 3 Results

### 3.1 Sequence analysis and identification of two transcript variants of *Iricα6*

A full-length mRNA sequence of the *α6* subunit of nAChR from *I. ricinus*, which we called *Iricα6*, has recently been sequenced (Rispe et al., 2022) and is available at NCBI (GenBank: MZ027288.1). The mRNA sequence of *Iricα6* has a total length of 3616 bp, comprising 318 bp of 5′ UTR (untranslated region), 1747 bp of 3′ UTR and an open reading frame (ORF) of 1551 bp, which corresponds to 517 amino acids. A BLASTP search of the NCBI database revealed that the protein sequence showed high similarity to nAChR α6/α7 subunits from other arthropod species, including *Ixodes scapularis* (97%), *Rhipicephalus microplus* (87%), *Bombyx mori* (96%), and *Drosophila eugracilis* (96%).

**Fig. 1:**
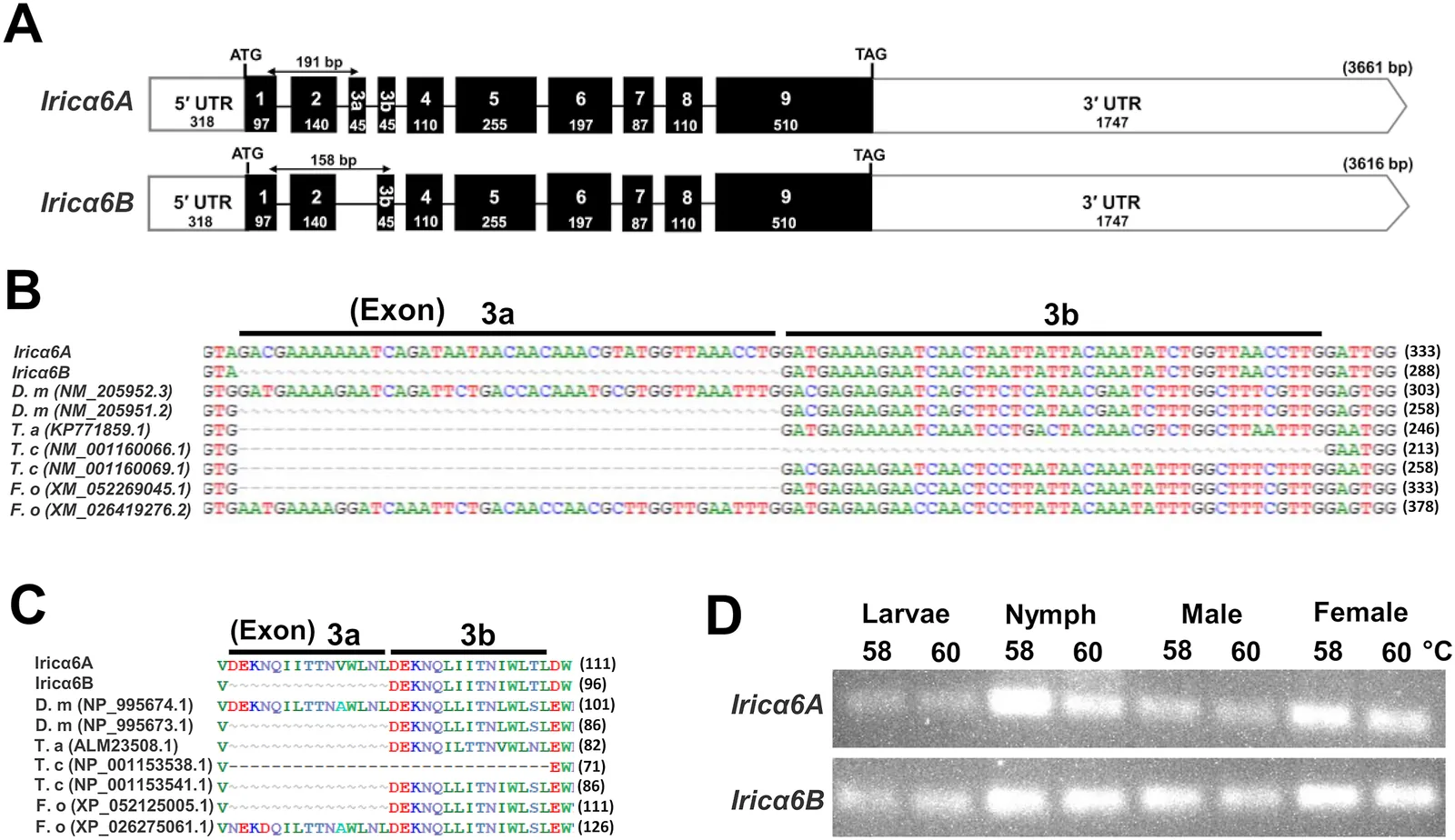
The organization of the *Iricα6* transcripts and RT-PCR analysis. **A** Genetic structure of the two variants of *Iricα6*. Exons are shown as black boxes and are scaled. Introns are shown as black lines and do not represent the original length. The untranslated regions (UTRs) are shown as white boxes. *Iricα6A* has both alternative exons 3a and 3b. *Iricα6B* has only 3b. The numbering of the exons is based on orthologs in *Drosophila melanogaster* and *Tribolium castaneum* (Grauso et al., 2002; Rinkevich and Scott, 2009). The length of each exon is shown in bp. Arrow lines indicate the positions of the primers designed to identify both variants of *Iricα6* in RT-PCR analysis. **B**, **C** Multiple alignments of nucleotide and amino acid sequences of alternative exon 3 from *I. ricinus* with its orthologs in other insect species. The NCBI accession numbers of the sequences used for alignment are provided in parentheses. **D** Amplification of both variants of *Iricα6* at different developmental stages of *I. ricinus* using variant-specific primers at annealing temperatures of 58 and 60 °C. Both *I. ricinus* transcript variants, with and without alternate exon 3a, were detected in larvae, nymphs, and adults. *Abbreviations*: *D. m*, *Drosophila melanogaster*; *T. a*, *Tuta absoluta*; *T. c*, *Tribolium castaneum*; *F. o*, *Frankliniella occidentalis*.

The complete open reading frame (ORF) of *Iricα6* was amplified through one-step RT-PCR using total RNA as a template. The amplified PCR product was cloned into a pT7TS-rich transcription vector. Multiple clones were sequenced, revealing two variants of *Iricα6* (Fig. 1A). One variant was 45 nucleotides longer than the *Iricα6* published sequence (Rispe et al., 2022) and was named *Iricα6A*. This variant had an ORF of 1596 bp and encoded a protein of 532 amino acids, with a predicted molecular weight of 59.9 kDa and an estimated pI of 5.61. The second variant possessed an ORF identical in size (1551 bp) to *Iricα6* and was named *Iricα6B*. This transcript encoded a 517-amino-acid protein with a predicted molecular weight of 58.2 kDa and an estimated pI of 5.70. The nucleotide sequences of both variants were submitted to NCBI under accession numbers PV393866 and PV393867. Both transcript variants were compared with the *I. scapularis* genome to examine exon and intron boundaries. Exons 1–9 were identical between the two transcript variants, except for exon 3 (Fig. 1A). Two alternative forms of exon 3 (3a and 3b) were evident, with both present in *Iricα6A*. In contrast, only alternative 3b was present in *Iricα6B* (Fig. 1A-C). This has previously been observed in insect species (Fig. 1B and C) and is attributed to alternative splicing (Jin et al., 2007; Shao et al., 2007; Rinkevich and Scott, 2009; Hou et al., 2014; Berger et al., 2016). Exon 3 is essential for receptor function and important for spinosad sensitivity. This has been observed in *Tuta absoluta*, where α6 transcripts from a spinosad-selected population lacked exon 3 as a result of exon skipping, and this alteration was associated with spinosad resistance (Berger et al., 2016).

Designing variant-specific primers that would allow accurate and comparable quantification of the two variants across different developmental stages (larvae, nymphs, and adults) of *I. ricinus* was technically challenging. Therefore, RT-PCR was performed on *I. ricinus* specimens. The results revealed the presence of both exon 3 forms (exon 3a and exon 3b) across the examined developmental stages. Two distinct primer sets, as shown in Fig. 1A, were designed to amplify alternative exon 3 with and without 3a (Fig. 1D).

### 3.2 Structural analysis

**Fig. 2:**
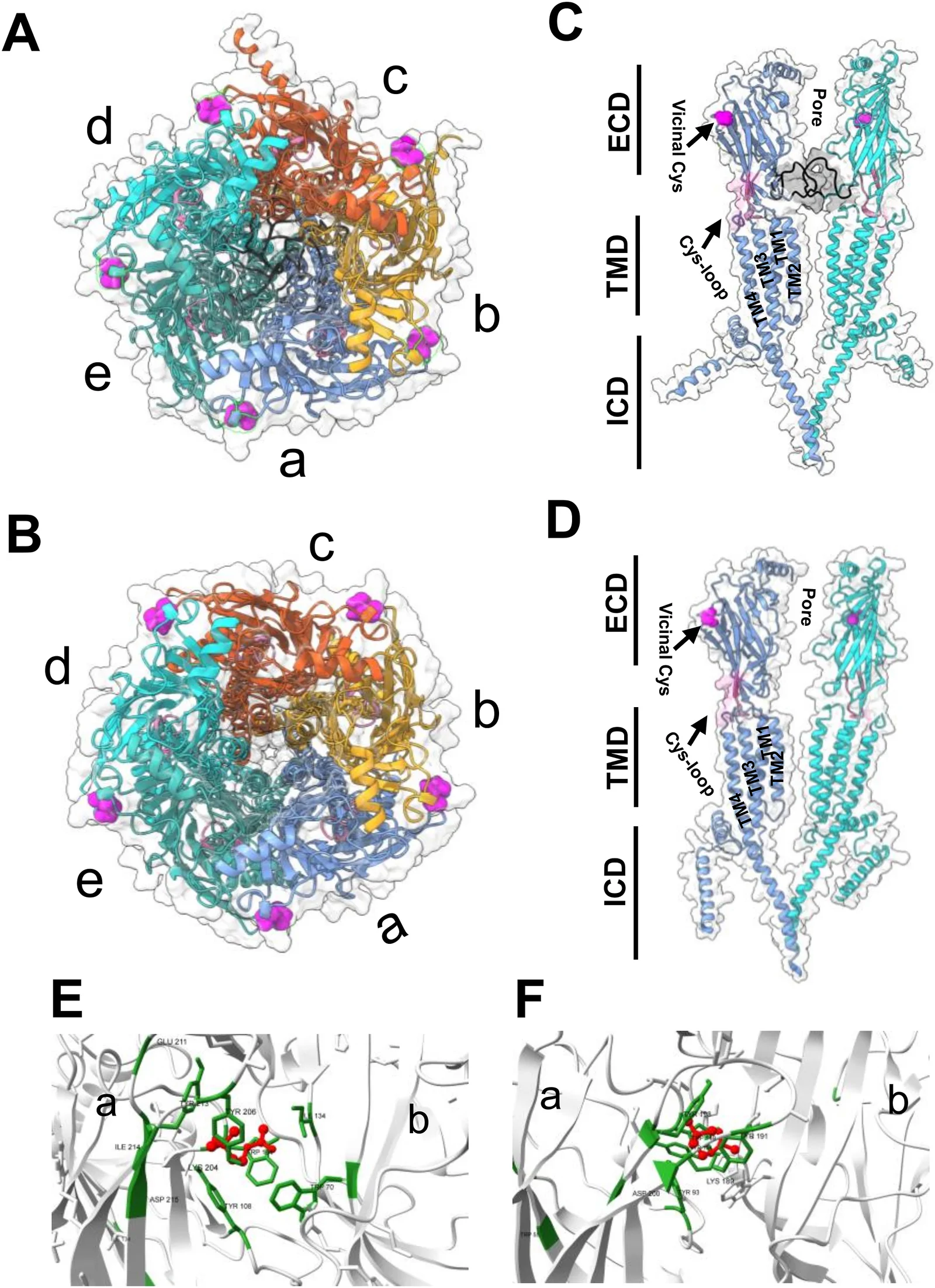
Structural analysis, pore detection and ACh binding sites of both variants of Iricα6. **A**, **B** Top view of the homopentamer of Iricα6A (**A**) and Iricα6B (**B**). Five identical subunits (a-e) are arranged around a pore. **C**, **D** Side view of the pore pathway of the variants Iricα6A (**C**) and Iricα6B (**D**) from the start to the end of the receptor. For clarity, only two subunits (a and b) are shown, with subunit a depicted in light blue and subunit b in cyan. The two cysteine residues in the Cys-loop are shown as magenta spheres, and the Cys-loop is highlighted in pink. A 15-amino-acid loop (black) was predicted within the pore pathway of variant Iricα6A (**C**), which is part of exon 3a and is absent in Iricα6B (**E**). The binding of ACh to the binding pocket of Iricα6A (**E**) and Iricα6B (**F**) at the interface between the a and b subunits was predicted using DockingServer. ACh is highlighted in red, and neighbouring residues are shown in green.

The tertiary structures of both variants of Iricα6 were predicted with standalone versions of AlphaFold2 and AlphaFold-Multimer. The homopentamers of both Iricα6 variants (Fig. 2A and B) were composed of five identical subunits (a-e) arranged around a central pore. Multiple protein sequence alignments (Supplementary Fig. S1) and predicted structures (Fig. 2C and D) of both variants exhibited features typical of nAChR subunits, including a N-terminal extracellular domain (ECD) with six loops (A-F) essential for ligand binding, two vicinal cysteines in loop C only found in alpha subunits (Kao et al., 1984), a C-terminal transmembrane domain (TMD) with four transmembrane helices (TM1-TM4), with TM2 facing the pore of the receptor and an intracellular domain (ICD) with the intracellular loop between transmembrane helices TM3 and TM4. Moreover, a loop of 13 amino acids, known as the Cys-loop and formed by a disulfide bond between two cysteine residues, was found at the interface between the TMD and ECD.

Structural prediction of Iricα6A and Iricα6B revealed a notable structural difference between the two variants within the ECD. The fifteen amino acids of exon 3a (Fig. 1A-D) found only in variant Iricα6A formed a loop (highlighted in black) that was predicted to extend into the receptor pore (Fig. 2C). This loop was absent in Iricα6B (Fig. 2D). The potential influence of this predicted loop on receptor assembly or function remains unclear at this point.

Since ACh is a natural agonist of nAChRs, we hypothesized that these structural differences could affect how ACh docks to the receptor. Accordingly, ACh docking was performed for both variants using AutoDock 4 *via* DockingServer, which predicted that ACh binds to the same binding site and residues in Iricα6A (Fig. 2E) and Iricα6B (Fig. 2F). The amino acids forming the ligand-binding pocket are shown in green, and acetylcholine in red. Complete details of the predicted amino acids forming the ligand-binding pocket are also provided in Supplementary Fig. S1.

### 3.3 Ancillary proteins are not essential for the functional expression of *Iricα6* in *X. laevis* oocytes

**Fig. 3:**
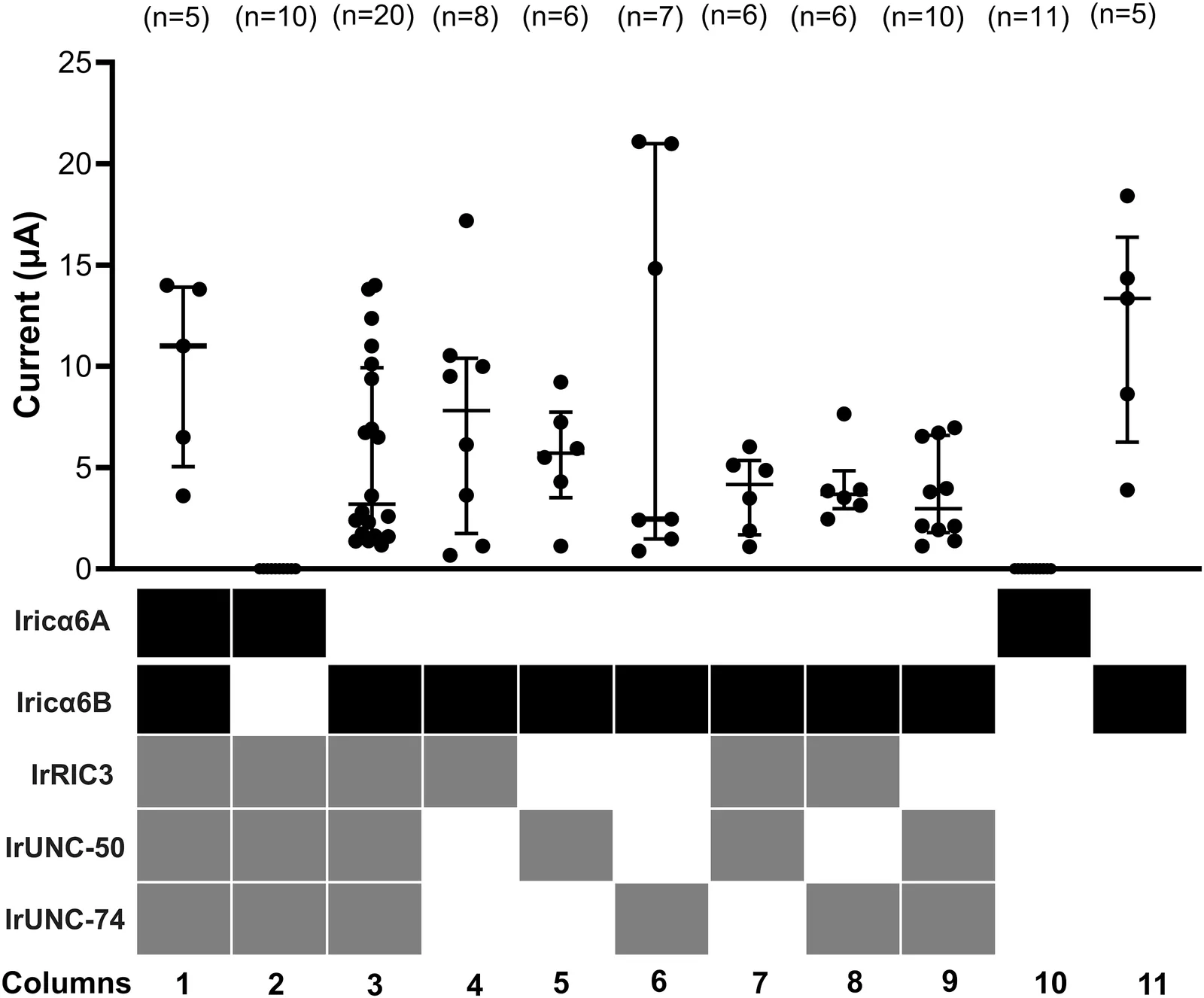
Response to ACh (300 μM) in *Xenopus laevis* oocytes expressing the two *Iricα6* variants, either separately or in combination, with or without ancillary proteins. The injection of Iricα6A and Iricα6B (black boxes), together with all three ancillary proteins (grey boxes), produced current (Column 1). The injection of *Iricα6A* alone, with or without ancillary proteins, failed to produce any measurable current (Columns 2 and 10) in response to acetylcholine (300 μM). In contrast, injecting *Iricα6B* alone in the presence of one or several ancillary proteins generated a current with 300 μm acetylcholine (Columns 3–9). Interestingly, injection of Iricα6B alone, without ancillary proteins, generated a current when exposed to 300 μM acetylcholine (Column 11). The number of oocytes used for each injection combination is indicated above the columns. A black dot represents individual oocyte recordings, and a horizontal bar represents the median ± interquartile range.

Two variants of *Iricα6* were expressed in oocytes by injecting the cRNA encoding each variant either alone or in combination, in the presence or absence of one, two or all three tick-specific ancillary proteins (RIC3, UNC-50 and UNC-74). Three days after the cRNA injections, the presence of functional nAChRs was investigated using the two-electrode voltage-clamp technique in the Roboocyte2 system. The co-injection of both variants (black boxes) together with all ancillary proteins (grey boxes) produced currents in response to 300 μM ACh (Fig. 3, Column 1; median: 11.00 μA; 95% CI: 4.076–15.484 μA). Surprisingly, cRNA injection of *Iricα6A* together with all ancillary proteins produced no currents (Fig. 3, Column 2), whereas injection of *Iricα6B* together with all ancillary proteins produced currents (Fig. 3, Column 3; median: 3.2 μA, 95% CI: 3.46026–7.77604 μA). Moreover, Iricα6*B* produced currents in response to 300 μM ACh even when cRNA for the ancillary proteins was excluded from the injection mixture, either individually or entirely (Fig. 3, Columns 4-9 and 11). No currents were generated when Iricα6*A* was injected in the absence of ancillary proteins (Fig. 3, Column 10). These results showed that Iricα6B can form a functional homomeric receptor in *X. laevis* oocytes in the absence of ancillary proteins.

### 3.4 Response of Iricα6B to different agonists

**Fig. 4:**
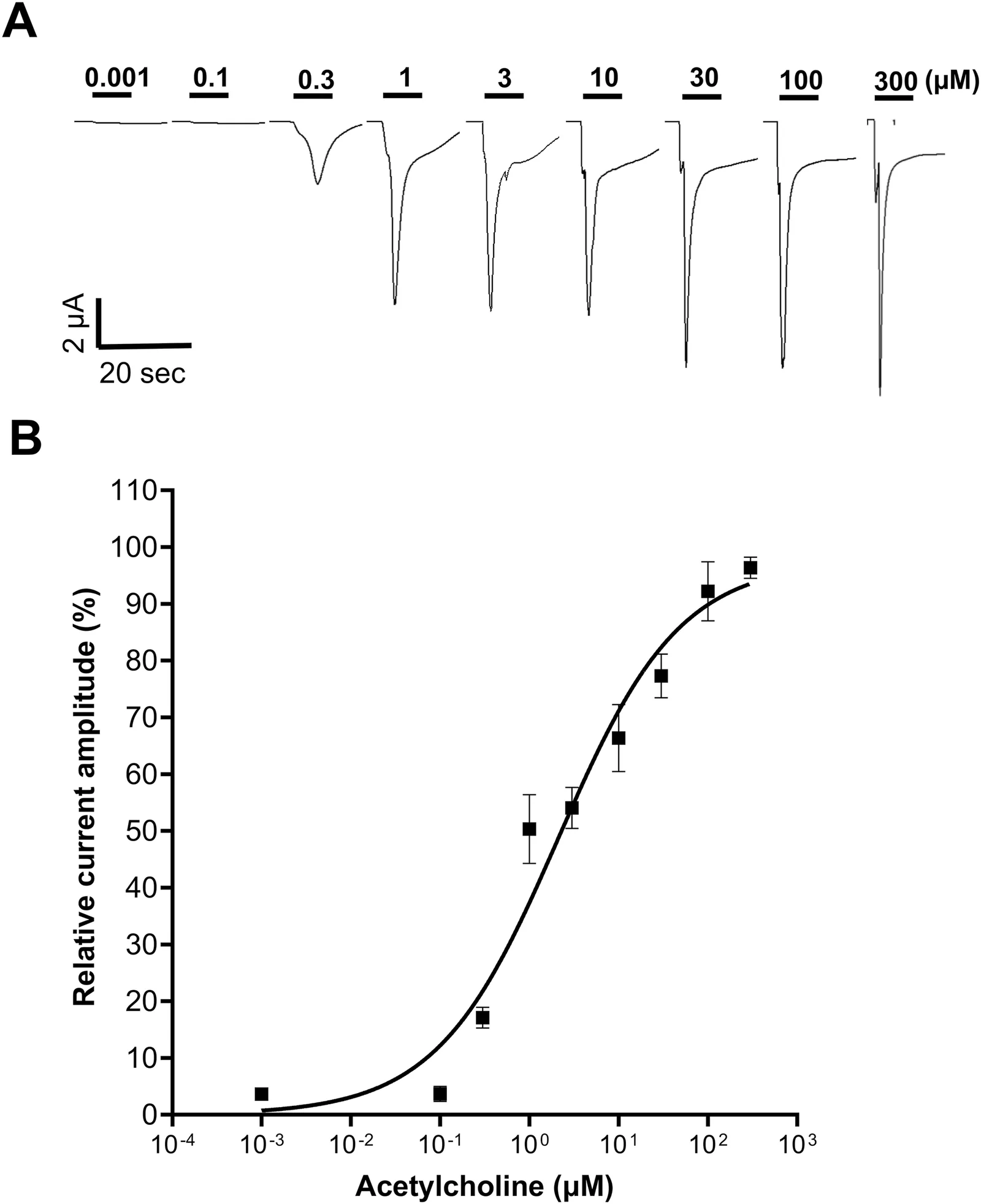
Representative current traces and dose-response curve of ACh on Iricα6B expressed in *Xenopus laevis*. **A** Representative current traces evoked by various μM concentrations of ACh in a single oocyte. The ACh exposure periods (7 s) are indicated by the short bars beneath the concentrations. **B** Dose-response curve of the Iricα6B receptor following exposure to increasing concentrations of ACh (μM). Each point represents the mean ± SEM (*n* = 7 for each concentration). Oocytes injected with water showed no response to ACh (not shown).

Oocytes were injected with Iricα6*B* cRNA, and 3 days later, the response to the natural agonist ACh was assessed. The application and removal of ACh activated and deactivated the Iricα6B receptor channel, as expected, and the response was concentration-dependent (Fig. 4). The estimated ACh EC_50_ from the dose-response curve (Fig. 4B) was 2 μM (0.6163 Hillslope, 95% CI, *n* = 7).

**Fig. 5:**
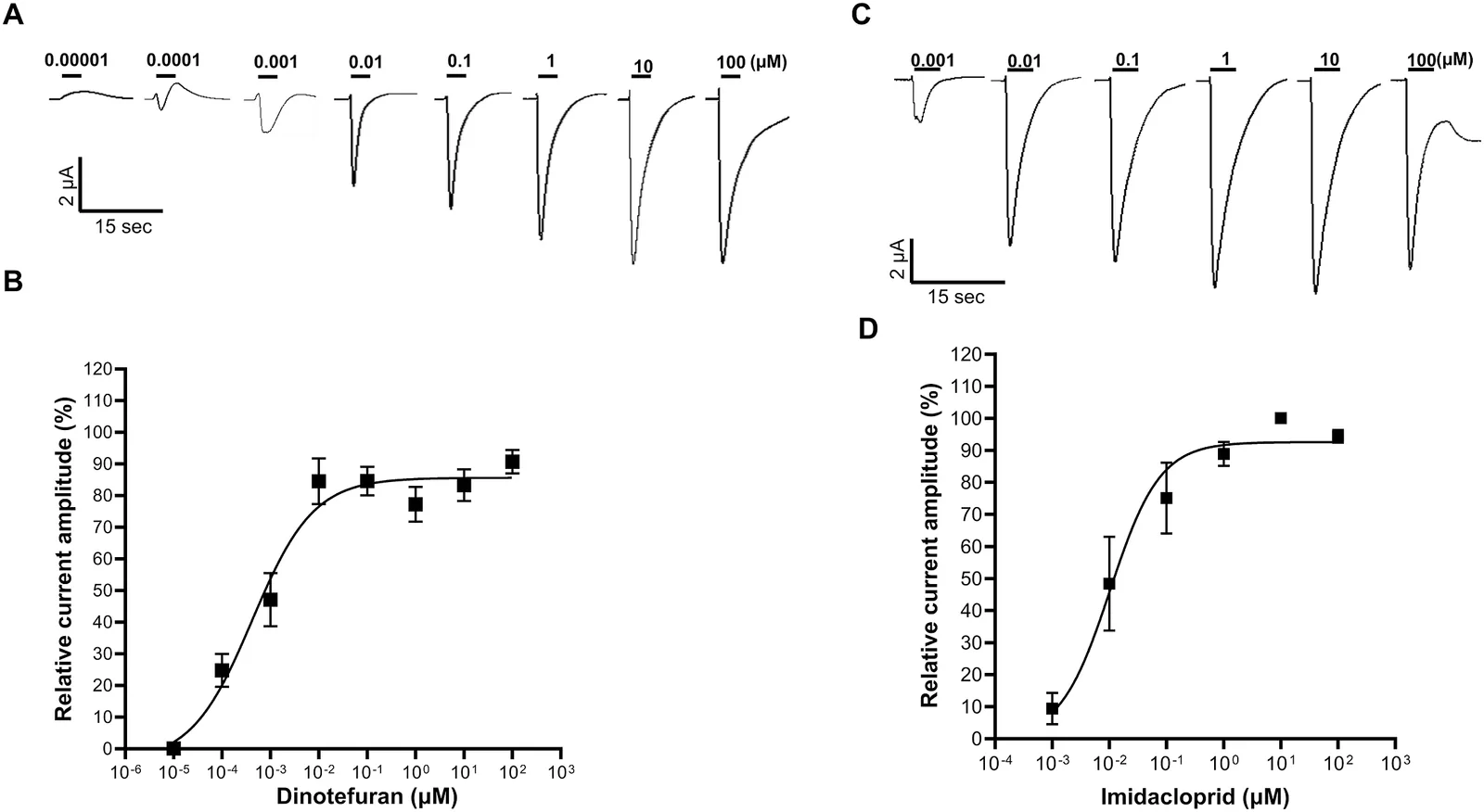
Representative traces and dose-response curves of two neonicotinoids on the Iricα6B receptor expressed in *Xenopus laevis*. Representative current traces from a single oocyte microinjected with *Iricα6B* cRNA and perfused with increasing concentrations (μM) of dinotefuran (**A**) and imidacloprid (**C**). The ACh exposure periods (7 s) are indicated by the short bars underlining the concentrations. Dose-response curves for dinotefuran (**B**) and imidacloprid (**D**) were obtained for the Iricα6B functional receptor. Each point represents the mean ± SEM for at least seven oocytes (dinotefuran) and four oocytes (imidacloprid). Oocytes microinjected with water showed no current traces when both neonicotinoids were applied.

**Fig. 6:**
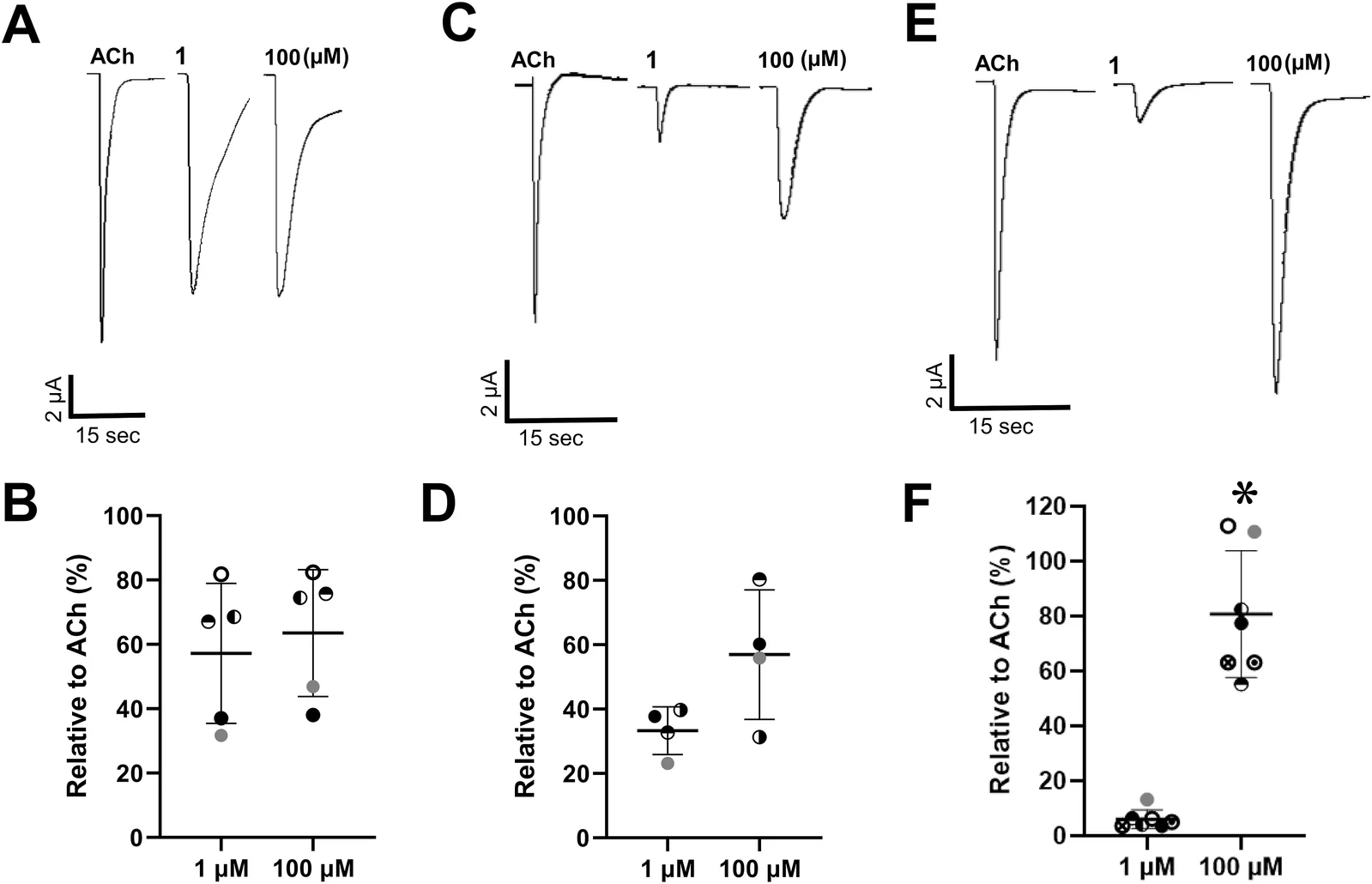
Representative current traces and the response of Iricα6B to nicotine and neonicotinoids. The traces shown in the upper panel were retrieved from a single oocyte after sequential application of ACh (300 μM) and two concentrations (1 and 100 μM) of nicotine (**A**) thiacloprid (**C**) and nitenpyram (**E**). Scatter plots show the response of the Iricα6B receptor to different concentrations of nicotine (**B**), thiacloprid (**D**) and nitenpyram (**F**) relative to ACh. Data are represented as the mean ± SD. The number of oocytes tested for each compound is indicated by dots, with the same dot colour or style indicating the same oocyte tested at two different concentrations of the same compound. Asterisks denote the statistical significance (Wilcoxon signed-rank test, *P* < 0.05).

Two neonicotinoids, dinotefuran and imidacloprid, were also tested on *X. laevis* oocytes expressing the Iricα6B receptor (Fig. 5). Iricα6B receptors showed high responses to dinotefuran and imidacloprid at doses as low as 0.001 μM. A slight increase in inward current was observed following perfusion with 0.01 μM of each neonicotinoid. Estimated EC_50_ values based on the dose-response curves generated for dinotefuran (Fig. 5B) and imidacloprid (Fig. 5D) were 0.42 nM and 11.6 nM, respectively. Relative to the ACh response, the maximum response peaks were 112.3% and 81% for dinotefuran and imidacloprid, respectively. Furthermore, responses to two concentrations (1 and 100 μM) of nicotine and two additional neonicotinoids (nitenpyram and thiacloprid) were tested in separate oocytes. Representative current peaks for nicotine, nitenpyram and thiacloprid, and the maximum amplitudes related to an initial exposure of ACh (300 μM), are depicted in Fig. 6. Nicotine was found to be very effective at a dose of 1 μM; however, the current did not change significantly when 100 μM was applied (Fig. 6A). There was no statistically significant difference in the maximum amplitude of the two nicotine concentrations compared to a fixed ACh concentration (Fig. 6B). Thiacloprid at concentrations of 1 μM and 100 μM showed a notable difference in current peaks, as shown in Fig. 6C. The mean maximum currents were 33% and 56% at concentrations of 1 μM and 100 μM, respectively, compared to ACh (Fig. 6D). The current peaks differed markedly at two different nitenpyram concentrations (Fig. 6E). At concentrations of 1 μM and 100 μM of nitenpyram, the mean maximum peak current was 6% and 81% compared to ACh for Iricα6B (Fig. 6F).

## 4 Discussion

In this study, the *Iricα6* subunit from *I. ricinus* was investigated at the molecular level and functionally in *X. laevis* oocytes. Two transcript variants were detected at all examined life stages of *I. ricinus*. Further, our results confirmed that one of the two identified Iricα6 variants forms a functional homomeric receptor in the absence of the tested ancillary proteins. This aligns with the expression of homomeric nAChRs in vertebrates (Schapira et al., 2002; Taly et al., 2009), which contrasts with heteropentameric receptors that rely on ancillary proteins to form functional receptors (Lees et al., 2014; Rufener et al., 2020).

The predicted structures of both variants of the Iricα6 receptor confirmed the presence of key features of nAChRs (Unwin, 2005), including six loops (A-F) in the ECD, four helixes in the TMD, with TM2 oriented towards the receptor pore, and a long loop between TM3 and TM4 in the ICD (Fig. 2C and D and Supplementary Fig. S1). However, an interesting structural difference was evident between the two variants of *Iricα6*, involving exon 3, which was found in two forms, 3a and 3b. Both receptor variants contained exon 3b; however, exon 3a was found only in *Iricα6A*, the variant that failed to form a functional receptor in the *X. laevis* oocyte expression system. Similar variants of exon 3 have been previously reported in various arthropods, including *D. melanogaster* (Grauso et al., 2002), *B. mori* (Shao et al., 2007) and *Tribolium castaneum* (Rinkevich and Scott, 2009). A higher prevalence of exon 3b was observed in the susceptible strain in contrast to the resistant strain, where 3a is most prevalent (Baxter et al., 2010).

Structural prediction of the Iricα6A pentamer (Fig. 2A and B) or single subunit (Fig. 2C and D) using AlphaFold2 revealed a 15-amino acid loop encoded by exon 3a, positioned on the same side as transmembrane helix 2 (TM2), which is essential for the formation of the nAChR ion channel pore. Docking analysis was performed on both variants of Iricα6 to identify the ACh-binding sites. The docking results predicted that both variants shared common ACh-binding pockets and residues across all six ACh-binding loops in the extracellular domain (ECD) (Fig. 2E and F, Supplementary Fig. S1), as previously described (Schapira et al., 2002; Taly et al., 2009; Mallipeddi et al., 2013). Based on these findings, we speculate that the apparent lack of response of Iricα6A to ACh in the *X. laevis* expression system may be associated with the loop encoded by exon 3a, which, based on structural prediction, could potentially interfere with the channel function. However, this hypothesis requires further investigation, including receptor assembly and trafficking as well as structural studies such as X-ray crystallography or cryo-EM.

Using *X. laevis* oocytes as an expression system, we demonstrated that injection of *Iricα6A* with or without ancillary proteins (RIC3, UNC-50, and UNC-74) did not produce any measurable current in response to the natural agonist ACh (Fig. 3). However, injecting the *Iricα6B* variant alone, with or without ancillary proteins, generated currents when exposed to ACh (Fig. 3) as well as to exogenous agonists (Fig. 4, Fig. 5, Fig. 6). This demonstrated that the Iricα6B variant can form functional homomeric receptors and that this can be achieved in the absence of ancillary proteins. Previous studies have shown that the functional expression of heteromeric nAChRs always requires ancillary proteins, whereas this is not always the case with homomeric nAChRs. For example, RIC3 is essential for the folding, assembly, and functional expression of various nAChR subtypes (Millar, 2008). Additionally, UNC-50 and UNC-74 (also known as TMX 3), together with RIC3, are essential for receptor formation and function, such as the Dα1/Dβ1 nAChR in *D. melanogaster* (Ihara et al., 2020) and nAChRs in the salmon louse *L. salmonis* (Rufener et al., 2020). The Iricα6B receptor responded to ACh concentrations ranging from 0.001 μM to 300 μM, with the maximum response at 300 μM. The calculated EC_50_ for ACh was 2 μM, which is higher than that of the homomeric α6-type nAChR from *Apis mellifera* (EC_50_ = 0.88 μM) (Hawkins et al., 2022). However, this is generally lower than for homomeric receptors in other arthropods. For example, nAChRs from *D. melanogaster* containing either Dα5 or Dα7 subunits displayed EC_50_ values of 8.8 μM and 7.7 μM, respectively (Ihara et al., 2020), and the homomeric nAChR ACR-16 from *Ancylostoma ceylanicum* exhibited an EC_50_ of 20.64 μM for ACh (Kaji et al., 2020). These findings suggested that the EC_50_ obtained for the homomeric Iricα6B nAChR showed high sensitivity to ACh under the conditions tested. However, it should be noted that EC_50_ varies with experimental setup and receptor compositions.

Furthermore, we tested the sensitivity of the Iricα6B receptor to different neonicotinoids and nicotine in the *X. laevis* oocytes expression system. Our findings demonstrated that imidacloprid and dinotefuran were potent agonists on the Iricα6B receptor and elicited responses even at the lowest concentration tested, 0.001 μM. Generally, neonicotinoid insecticides are believed to elicit poor responses at tick nAChRs (Erdmanis et al., 2012). The study conducted by Le Mauff and colleagues showed that four tested neonicotinoids, including imidacloprid, have little effect on nAChRs expressed in tick synganglion membranes (Le Mauff et al., 2020) and therefore lack acaricidal activity. In another study by Lees et al. (2014), co-expression of the *R. sanguineus* α1 subunit with the β2 subunit resulted in robust responses to ACh and nicotine while being insensitive to imidacloprid. Our study showed that Iricα6B receptor expressed in *X. laevis* oocytes responded well to imidacloprid, with an estimated EC_50_ of 11.6 nM. Thus, although imidacloprid has been promoted as an insecticide rather than an acaricide (Jeschke and Nauen, 2008), we show that sensitivity at the receptor level is preserved in ticks, at least for Iricα6B. Variation in neonicotinoid sensitivity also occurs among insect receptors. For example, the estimated EC_50_ of imidacloprid in neuronal cells of antennal lobes in *A. mellifera* was 0.87 μM (Nauen et al., 2001), and pEC_50_ (negative logarithm of the EC_50_) for imidacloprid estimated in the terminal abdominal ganglion of American cockroaches was 5.9 (corresponding to an EC_50_ value of 1.3 μM) (Ihara et al., 2006). Contrary to these observations, the cockroach Pameα7 nAChR subunit, which forms a homomeric receptor when expressed in *X*. *laevis* oocytes, does not respond to imidacloprid (Cartereau et al., 2020, 2021). The sensitivity of nAChRs to imidacloprid in insects has been linked to arginine at position 81 (R81) in loop D of the β1 subunit (Erdmanis et al., 2012). Moreover, mutation of R81 in the insect β1 subunit has been associated with high levels of resistance to imidacloprid and other neonicotinoids (Bass et al., 2011; Hirata et al., 2015). In several tick species, arginine has been replaced with glutamine (Q) in the β subunit (Erdmanis et al., 2012; Boussaine et al., 2025). Here, we have studied a homomeric receptor composed solely of α-subunits. Multiple alignment of Iricα6 protein sequences (Supplementary Fig. S1) showed that no arginine or glutamine was found in loop D, which could explain the high sensitivity of Iricα6B to imidacloprid in *I*. *ricinus*. However, this possibility remains unknown and requires further investigation using functional and mutational approaches. Our recent results further confirm that dinotefuran is more potent than imidacloprid, with an EC_50_ of 0.42 nM. Few studies have shown dinotefuran agonism at recombinant heteromeric or hybrid nAChRs from arthropods rather than at homomeric receptors. Dinotefuran acted as a full agonist when tested on the *D*. *melanogaster* SAD (α subunit) and chicken β2 hybrid nAChR (Kagabu et al., 2002). The activity of dinotefuran has also been characterized on *Nilaparvata lugens* α1 and rat β2 hybrid nAChR, with an estimated EC_50_ of 122 ± 6.9 μM (Liu et al., 2006). In contrast, the present study demonstrates the activity of dinotefuran on Iricα6B homomeric nAChR. Notably, the EC_50_ of 0.42 nM for dinotefuran was substantially lower than for previously characterized arthropod hybrid heteropentameric nAChRs.

To check if the receptor also responded to other compounds known to act on nAChRs, a small-scale experiment with two concentrations of nicotine, thiacloprid, and nitenpyram was conducted. Nicotine is reported to be an effective agonist of homomeric receptors, e.g. on *Pameα7* (Cartereau et al., 2020, 2021). Furthermore, a homomeric receptor from the nematode *Ascaris suum* (Asu-ACR-16) displayed similar EC_50_ values for nicotine and ACh, 4.5 μM and 5.9 μm, respectively (Abongwa et al., 2016). Interestingly, for Iricα6B, nicotine-induced inward currents were 57% and 63% of those induced by ACh at concentrations of 1 and 100 μM, respectively, indicating lower agonist efficacy at the concentrations tested. The responses to thiacloprid on Iricα6B were also tested at 1 and 100 μM (Fig. 6), yielding mean maximal currents of 33% and 56%, respectively, relative to ACh. This suggests that thiacloprid, along with nicotine, elicited weak responses. The response of Iricα6B to nitenpyram at 1 μM was lower than its response to nicotine and thiacloprid (Fig. 6); however, at 100 μM, the mean maximal peak current reached 81% of ACh, indicating that nitenpyram is more efficacious than nicotine and thiacloprid at this concentration. Nonetheless, because full dose-response curves were not obtained, some uncertainties remain regarding these ligands.

Altogether, our findings demonstrate that only the Iricα6B variant from *I. ricinus* can form homomeric receptors when expressed in *X*. *laevis* oocytes, and that this expression occurs independently of ancillary proteins. Furthermore, Iricα6B responded not only to ACh and nicotine but also to several neonicotinoids. These findings provide a basis for further investigation of the Iricα6 receptor as a potential target for the development and validation of acaricides.

## 5 Conclusions

This study presents the molecular and functional characterization of the α6 subunit of nAChR from *Ixodes ricinus* and identifies two alternative splice variants of the α6 subunit. Our RT-PCR analysis revealed that both variants were detected across all examined life stages of *I. ricinus*. Of the two variants, only Iricα6B formed a functional homomeric receptor when expressed in *X*. *laevis* oocytes, independent of ancillary proteins, whereas Iricα6A was non-functional under the tested conditions. Our results further demonstrated that Iricα6B receptor exhibited high sensitivity to ACh, nicotine and several neonicotinoids. These findings demonstrated Iricα6B as a pharmacologically responsive nAChR subunit in *I. ricinus* and provide new understanding of the molecular and functional diversity of tick nAChRs. Moreover, these findings support further investigation of this receptor as a potential molecular target for the development and evaluation of compounds for tick control.

## Ethical approval

All experiments involving *Xenopus laevis* oocytes were conducted under the procedures approved by the Norwegian Directorate of Health (approval no. 20/28865-2).

## CRediT authorship contribution statement

**Muhammad Tanveer Khan:** Conceptualization, Methodology, Formal analysis, Writing - original draft, Writing review & editing, Visualization, Validation. **Kiranpreet Kaur:** Writing - original draft, Writing review & editing. **Tor Einar Horsberg:** Conceptualization, Validation, Formal analysis, Data curation, Writing - review & editing, Supervision, Funding acquisition. **Marit Jorgensen Bakke:** Conceptualization, Validation, Formal analysis, Data curation, Writing - review & editing, Visualization, Supervision, Funding acquisition.

## Statement on the use of AI-assisted technologies

During the preparation of this manuscript, no AI-assisted tools were used to develop scientific content, data analysis or conclusions.

## Funding

This work was supported by the 10.13039/501100005416Research Council of Norway, Grant Number 325190.

## Declaration of competing interests

The authors declare that they have no known competing financial interests or personal relationships that could have appeared to influence the work reported in this paper.

## Data Availability

The data supporting the conclusions of this article are included within the article and its supplementary files. The newly obtained nucleotide sequences of the two variants of the α6 nAChR subunit have been deposited in the GenBank database under the accession numbers PV393866 and PV393867.
