# Supplementary material for: No ancillary proteins are essential for the functional heterologous expression of the homomeric α6 nicotinic acetylcholine receptor from the brown castor tick Ixodes ricinus
Source: Curr Res Parasitol Vector Borne Dis. 2026 Sep 4;10:100430. doi: 10.1016/j.crpvbd.2026.100430 (PMC13578988; doi:10.1016/j.crpvbd.2026.100430)
Supplement: Supplementary file 1 [file mmc1.pdf]

Supplementary materials

No ancillary proteins are essential for the functional heterologous expression of the homomeric α6 nicotinic acetylcholine receptor from the brown castor tick, *Ixodes ricinus*

Muhammad Tanveer Khan[a] *, Kiranpreet Kaur[a, b], Tor Einar Horsberg[a], Marit Jorgensen Bakke[a]

[a] *Norwegian University of Life Sciences, Faculty of Veterinary Medicine, Aas, Norway*

[b] *Aker Qrill Company, Lysaker, Norway*

* Corresponding author.

E-mail address: muhammad.tanveer.khan@nmbu.no (Muhammad Tanveer Khan)

Supplementary Table S1. Primers used in this study.

Primer name	Sequence (5'-3')	Purpose
**BmtI_Ir-UNC50-F**	ATTCGCTAGCACCATGGACAAGATGAGTACAGC	Cloning
**BsrGI_Ir-UNC50-R**	ATGGTGTACATTCACATGACCCGAAATTTGTAGAAG	
**ClaI_Ir-UNC74-F**	ATTCATCGATACCATGGGCGAAGTCTGTGTCAA	
**BsrGI_Ir-UNC74-R**	ATGGTGTACATTTACTCCTTCTTTTCGTGGCG	
**ClaI_Ir-RIC3s-F**	ATTCATCGATACCATGGCCACGCAGCTCG	
**AflII_Ir-RIC3s-R**	ATGGCTTAAGTTCAACAAGAGGCTCGCGTAC	
**BmtI_Ir-nAChRa6-F**	ATTCGCTAGCACCATGGGGAGGACGGCGCGATG	
**ClaI_Ir-nAChRa6-R**	ATGGATCGATTTACGCGACCAAGTGCGGTGC	
**Ir-nAChRa6-DF**	GCAAGATACGGAGCAAGGTG	RT-PCR (Both)
**Ir-nAChRa6-C2R**	CCAGGTTTAACCATACGTTTGTTG	Variant A
**Ir-nAChRa6-C3R**	GATTCTTTTCATCTACGTCGATAATCT	Variant B
**pT7Ts-rich Seq1_F**	TCCGGCTCGTATGTTGTGTG	Primers for template amplification for cRNA
**pT7TS_3'_R1**	GATCCTCTAGAGTCGACCTGCA	

Abbreviations: RT-PCR, reverse transcription polymerase chain reaction; cRNA, capped RNA.

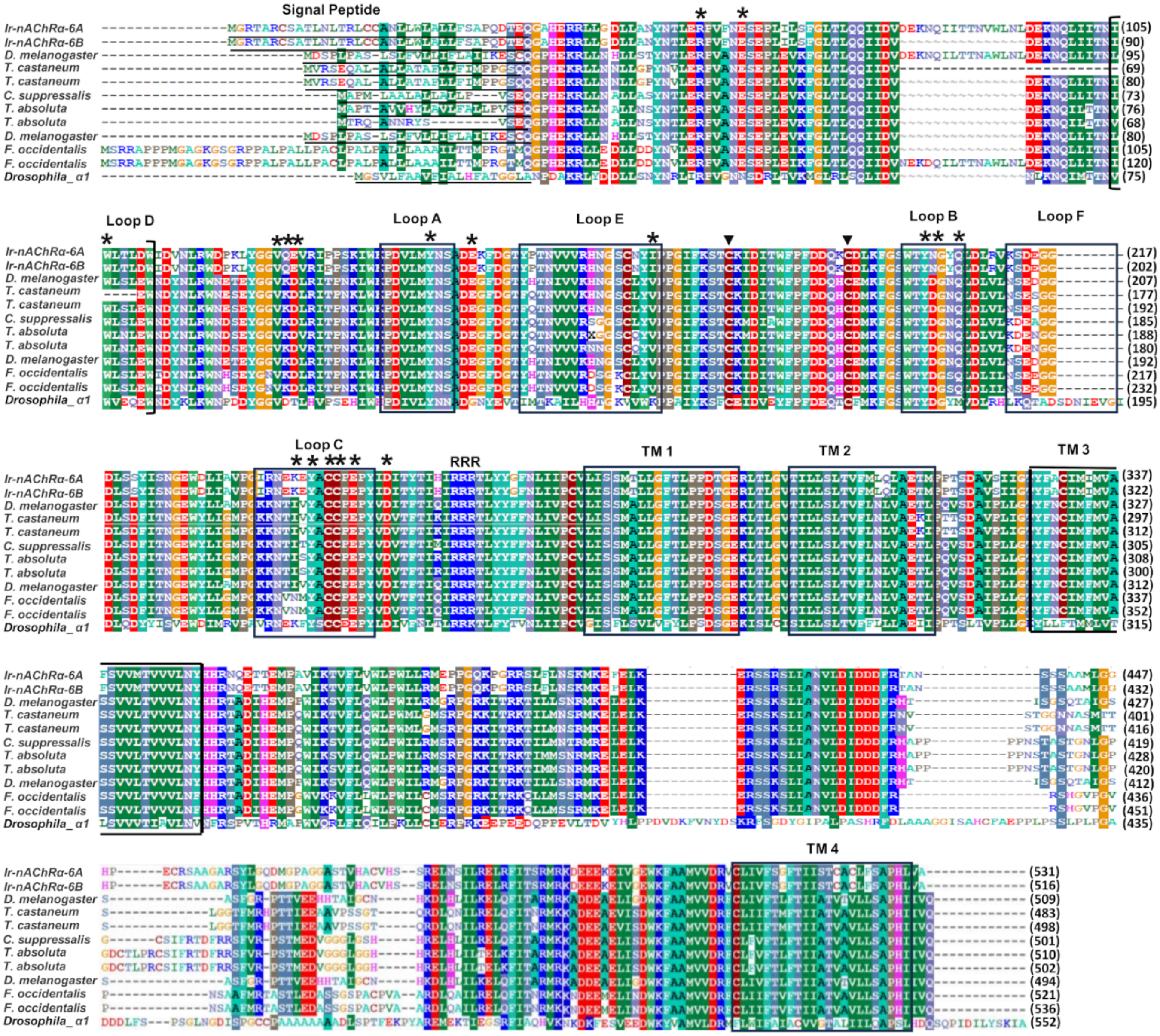

Supplementary Figure S1. Multiple alignments of nAChR subunits from different organisms. The multiple amino acid sequence alignment of two variants of the Iricα6 subunit with insects utilizes the α1 nAChR protein sequence of *Drosophila melanogaster* (CAA30172) for comparison. The N-terminal predicted sequences of signal peptides are underlined. Six loops (A-F) are indicated in boxes, with asterisks (*) representing the predicted acetylcholine binding residues alongside two cysteine residues in the C-loop. The ACh molecule binds between two subunits, where loops A, B, and C are referred to as the principal site, and loops D, E, and F are referred to as the complementary site. The sequence between the two arrowheads indicates the cys-loop. An RRR motif is displayed. Four transmembrane helices (TM1-TM4) are also shown in boxes.
